# The effect of opening window position on aerosol transmission in an enclosed bus under windless environment

**DOI:** 10.1063/5.0073171

**Published:** 2021-12-01

**Authors:** Feng Yao, Xiangdong Liu

**Affiliations:** 1 Jiangsu Key Laboratory of Micro and Nano Heat Fluid Flow Technology and Energy Application, School of Environmental Science and Engineering, Suzhou University of Science and Technology, Suzhou 215009, China; 2 College of Electrical, Energy and Power Engineering, Yangzhou University, Yangzhou, Jiangsu 225009, People's Republic of China

## Abstract

The potential risk of spreading a virus during bus transportation motivates us to understand the aerosol transmission of SARS-CoV-2 and seek effective ways to protect passengers in a bus. In this paper, a typical scenario in which the virus spreads in a bus under a windless environment is numerically studied for further understanding of the spreading characteristics of aerosol transmission in an enclosed space. The air flow in the bus and the spreading processes of droplets with different open windows configurations are obtained and analyzed. The variations of droplet concentration in the air with time are examined and analyzed. In addition, the transient droplet concentration deposited on the passengers is also counted to analyze the potential contact transmission. The results indicate that opening a window next to an infected person shows an unsatisfactory performance in limiting droplet spreading range and reducing droplet concentration, eventually leading to a high risk of infection by aerosol transmission following contact transmission. In addition, opening multiple windows also shows an unsatisfactory result for removing droplets in a bus since the turbulence flow accelerates the spreading speed and expands the spreading range. In contrast, the droplets are removed from the indoor space of the bus quickly if a window is opened in the row in front of the infected person, which is beneficial for reducing aerosol and contact transmission in the bus. Furthermore, it is strongly recommended to avoid sitting in the row in front of the infected person where the highest droplet concentration can be observed.

## I. INTRODUCTION

Due to the high infectivity (the median value of the basic reproduction number is approximately 5.71^1^) and high concealment (the median incubation period is approximately four days) of coronavirus disease 2019 (COVID-19) caused by a novel virus of the β-coronavirus genus (SARS-CoV-2), COVID-19 has spread around the world rapidly since the end of 2019, accompanied by insufficient attention paid to this virus by countries all over the world and few preventative and control measures taken at the beginning of the epidemic. Based on statistics from the coronavirus resource center at Johns Hopkins University (data up to September 26, 2021), over 230 × 10^6^ global cumulative cases have been confirmed and more than 4.7 million patients have died. In addition, there are still more than 30 × 10^6^ global active cases of COVID-19, and over 0.5 × 10^6^ new daily cases being confirmed since November 2020. Therefore, how to prevent and control the spread of COVID-19 remains an urgent issue worldwide although the vaccination has been carried out on a large scale.

According to recent research, it is generally acknowledged that there are four main transmission routes for SARS-CoV-2, including contact, fecal-oral transmission, droplets, and aerosols.^2^ The widespread transmission of COVID-19 is most likely to occur through droplet and aerosol transmission routes.^3^ Thousands of droplets containing SARS-CoV-2 can be sprayed into the air by the coughing or sneezing of a virus carrier.^4^ Droplets with large diameters will fall onto the surface of objects within 6 ft distance because of gravity forces, causing subsequent contact infection.^5^ Droplets with small diameters will float in air-forming aerosols since the Brownian force is equivalent to gravity.^6^ This can lead to a dramatically expanded transmission range, and people can be very susceptible to infection by breathing in air that contains the virus.^7^ Therefore, wearing a mask and avoiding places where crowds gather are suggestions emphasized by doctors to prevent cross infection.^8^

Despite these methods, sometimes people can find themselves in a relatively closed space, such as taking public transportation,^9^ dining in a restaurant,^10^ and hospitals.^11^ In these cases, SARS-CoV-2 can be spread in an enclosed space if an infected person spends time in this place. Li *et al.*^12^ revealed an alarming result that the upward movement of numerous virus particles created by flushing a toilet seat can reach higher than 1 m, leading to large-scale virus transmission. Inspired by this surprising conclusion, we believe that it is important to understand the spreading characteristics of droplets with virus in an enclosed space, which can provide useful guidance for minimizing the risk of cross-infections. Busco *et al.*^13^ predicted the spread range of a human sneeze by computational fluid dynamics (CFD) simulation combined with a discrete phase model (DPM), and the results were in good agreement with the experimental data. Wang's group proposed a ward air pattern optimization method based on an analysis of airflow organization and the movement characteristics of particles.^14^ A CFD simulation of droplet motion characteristics with air flow can provide useful information for predicting the spreading characteristics of droplet and aerosol transmission. So far, many studies have been conducted to analyze the transmission routes and prevention methods of COVID-19,^15–17^ and many of them have investigated the effect of air flow on droplets and aerosol transmission with the assistance of the CFD method. Xia *et al.*^18^ pointed out that the regular cleaning of the bus is necessary as a considerable number of droplets would deposit on the surface of objects in the bus. Zhu *et al.*^19^ considered that the use of filtration systems will effectively reduce the exposure of the public to the airborne virus based on the simulation results. Mathai *et al.*^20^ found that the airborne transmission of the virus in a moving car will not always be suppressed by opening all the windows.

As seen, most of the existing literature studies are focused on the analysis of the aerosol transmission in moving vehicles, which is indeed very important and necessary for the prevention in public traffic. However, it should be noted that, different from the promoted ventilation of the help of ambient wind pressure in a moving bus by opening windows, the air conditioning ventilation is the main method for strengthening air flow in a waiting bus. However less attention is paid on the virus diffusion in a bus during the period between boarding and moving (usually 5–30 min), which is short for passengers but is long enough for virus infection. Therefore, in this paper, the enclosed space in a bus is chosen as a virus transmission location, the influence of window-opening positions on air flow organization and droplet movement is analyzed, and the dynamic spreading characteristics of droplets, including the distribution of droplets with time and the transient deposition amount on passengers, are also discussed to provide guidelines for reducing cross-infections when taking public transportation.

## II. MODEL DESCRIPTION

### A. Geometry of the bus

**FIG. 1.**
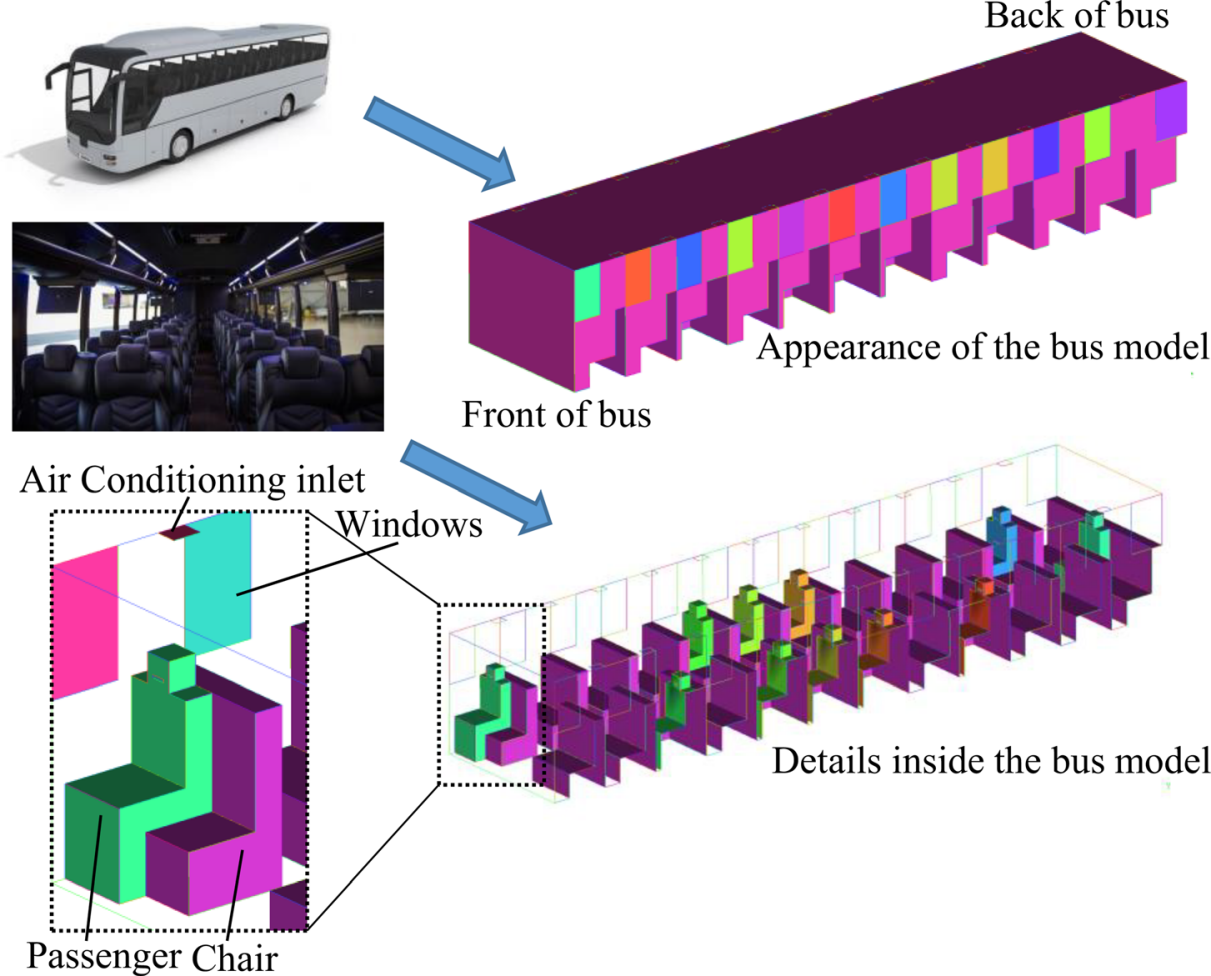
Schematic of a 3D enclosed bus with passengers.

**FIG. 2.**
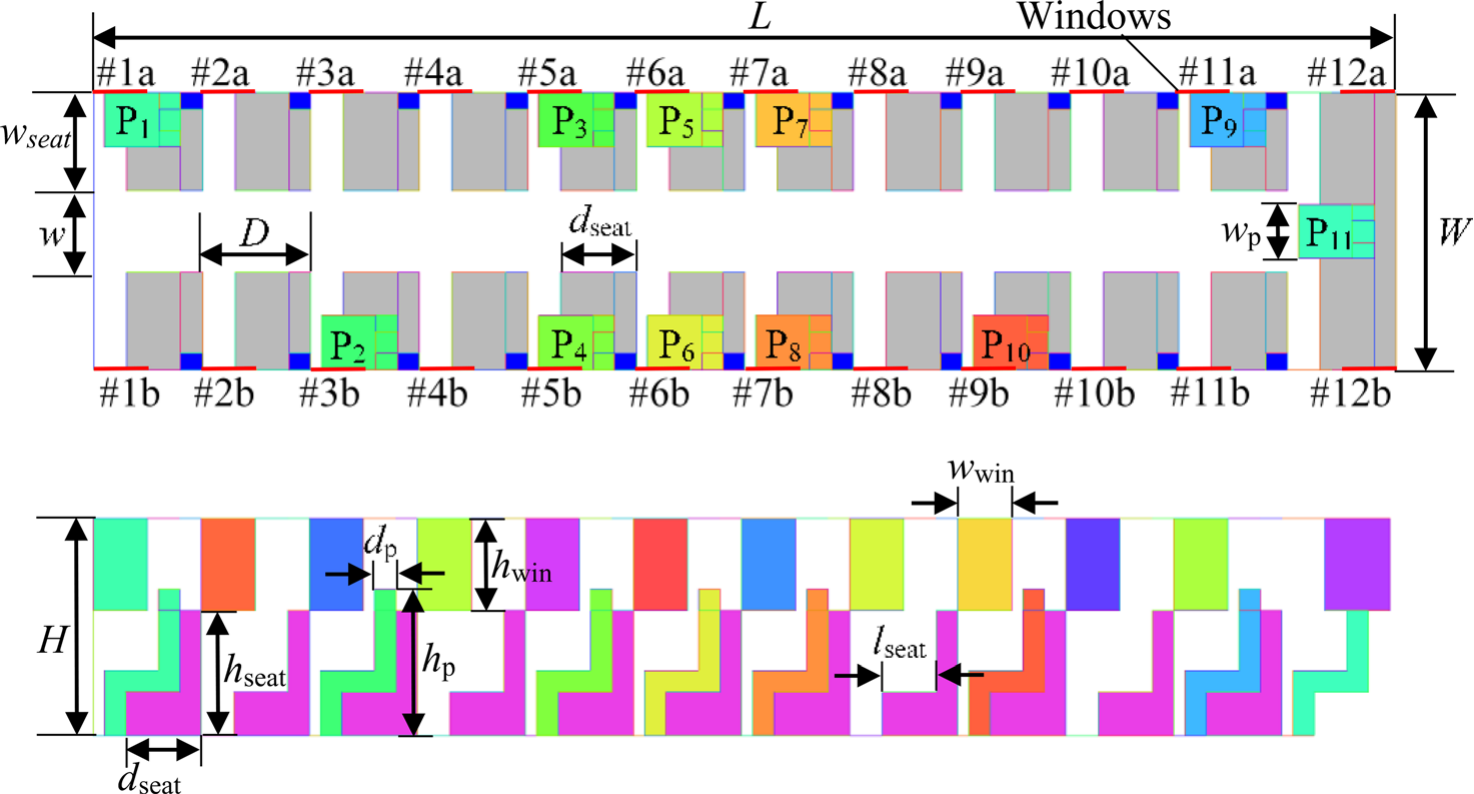
Distributions of passengers in the enclosed bus.

As shown in Fig. 1, a simplified geometric model of a bus with dimensions of 12 × 2.55 × 2 m^3^ (length × width × height) is built for simulation. The bus contains 12 rows of seats, 24 windows (Nos. 1a–12a on the left side and Nos. 1b–12b on the right side), and 22 air conditioning inlets. There is a 75 cm wide aisle in the middle of the first 11 rows of seats. The seat is 90 cm long (except for the last row, which is 255 cm long), 50 cm wide, and 40 cm high, while its back is 90 cm long, 20 cm wide, and 115 cm high, which is the same height as the passenger's shoulder. Twenty windows are 26 cm wide and 85 cm high. The remaining two windows at the back of the bus are 60 cm wide and 85 cm high. All of the windows are closed by default unless specified. In order to investigate the aerosol transmission from the patient to the other passengers, the passengers are placed around the patient, which may be different from the actual situation that the passengers keep distance while sitting. In addition, some other passengers are placed as far away from the patient as possible to investigate the aerosol transmission range. Eleven passengers (P1–P11) represented by several cuboid blocks are assumed to sit dispersedly in the bus carriage, and their positions are marked in Fig. 2, which are the typically positions around the infected person. To simulate the sneezing process, a mouth with a width of 10 cm and height of 2 cm is set on the head of every passenger. The detailed parameters of the geometric model are listed in Table I.

**TABLE I.** Parameters of the bus and passengers.

Parameter	Description	Dimension
*L* (m)	Length of vehicle	12
*W* (m)	Width of vehicle	2.55
*H* (m)	Height of vehicle	2
*w* (m)	Width of aisle	0.85
*D* (m)	Distance between rows	1
*l*_seat_ (m)	Length of seat surface	0.5
*d*_seat_ (m)	Total length of seat	0.7
*w*_seat_ (m)	Width of seat	0.9
*h*_seat_ (m)	Height of seat	1.15
*w*_win_ (m)	Width of window	0.5
*h*_win_ (m)	Height of window	0.85
*h*_p_ (m)	Height of passenger	1.35
*d*_p_ (m)	Thickness of passenger	0.2
*w*_p_ (m)	Width of passenger	0.5

### B. Mathematical model

In this paper, the droplets enter the indoor environment quickly and with high speed caused by sneezing and move with the air flow. Therefore, a turbulence model and discrete phase model are both utilized in the simulation.

#### 1. Turbulence model for the air flow

When sneezing occurs, air containing thousands of droplets are sprayed out from the mouth at a speed of ∼50 m/s in less than 1 s.^21^ In this context, the air flow should be treated as turbulent flow when the Reynold number is larger than 13 000. Therefore, the realizable *k*–*ε* model, which has been successfully utilized to predict the particle movement caused by toilet flushing,^14,21^ is adopted in this paper. The turbulent kinetic energy *k* and dissipation rate *ε* can be solved by the following governing equations:

$$\frac{\partial }{\partial t}\left(\rho k\right)+\frac{\partial }{\partial x_{i}}\left(\rho ku_{i}\right)=\frac{\partial }{\partial x_{i}}\left[\left(\mu +\frac{\mu_{t}}{\sigma_{k}}\right)\frac{\partial k}{\partial x_{i}}\right]+G_{k}+G_{b}-\rho \varepsilon -Y_{M}+S_{k},$$ (1)

$$\frac{\partial }{\partial t}\left(\rho \varepsilon \right)+\frac{\partial }{\partial x_{i}}\left(\rho \varepsilon u_{i}\right)=\frac{\partial }{\partial x_{i}}\left[\left(\mu +\frac{\mu_{t}}{\sigma_{\varepsilon }}\right)\frac{\partial \varepsilon }{\partial x_{i}}\right]+\rho C_{1}S\varepsilon -\rho C_{2}\frac{\varepsilon^{2}}{k+\sqrt{v}\varepsilon }+C_{1\varepsilon }\frac{\varepsilon }{k}C_{3\varepsilon }G_{b}+S_{\varepsilon },$$ (2)

where *μ*_t_ = *ρC_μ_k*2/*ε* is the turbulent viscosity and *C_μ_* represents the turbulence fields. *σ_k_* = 1.0 and *σ_ε_* = 12 are the turbulent Prandtl numbers for *k* and *ε*, respectively. *G_k_* and *G_b_* are the generation of turbulence kinetic energy due to the mean velocity gradients and buoyancy force and can be calculated by

$$G_{k}=-\bar{\rho {u_{i}}^{\prime }{u_{j}}^{\prime }}\frac{\partial u_{j}}{\partial x_{i}},$$ (3)

$$G_{b}=\rho g\frac{\mu_{t}}{Pr_{t}}\frac{\partial T}{\partial x_{i}}.$$ (4)

*Y*_M_ = 2*ρεM_t_*2 is the fluctuation effect on the overall dissipation rate in compressible turbulence. *M*_t_ = (*k*/a^2^)^1/2^ is the turbulent Mach number. *C*_1_ = max [0.43, *η*/(*η* + 5)], *C*_2_ = 1.9, and *C*_1__*ε*_=1.44. *C*_3__*ε*_ is the buoyancy effect on ε. *S_k_* and *S*ε are source terms.

#### 2. Discrete phase model (DPM) for droplets

In the DPM method, the movement of particles is determined by the combined effect of drag force, buoyancy, gravity, and Brownian force. The force balance of a particle in the Lagrangian reference frame can be given by

$$\frac{d{\vec{u}}_{p}}{dt}=F_{D}\left(\vec{u}-{\vec{u}}_{p}\right)+\frac{g\left(\rho_{p}-\rho \right)}{\rho }+\vec{F},$$ (5)

where $\vec{u}$ is the velocity of air, ${\vec{u}}_{p}$ is the velocity of a single droplet, *ρ* is the density of air, *ρ*_p_ is the density of the droplet, and $\vec{F}$ is the acceleration caused by additional forces such as Brownian force. *F*_D_ ( $\vec{u}-{\vec{u}}_{p}$) is the drag force, and *F*_D_ can be calculated by

$$F_{D}=\frac{18\mu }{\rho_{p}d_{p}^{2}}\frac{C_{D}Re}{24},$$ (6)

where

CD=0.424,Re>1000,24Re1+16Re2/3,Re≤1000, Re=ρdpu→p−u→μ.

### C. Computational model

#### 1. Assumptions

Some assumptions have to be premade after considering the computational cost and complexity of simulating the sneezing process in an enclosed bus:

(1) The heat and mass transfer between droplets and air is neglected during the simulation.
(2) The merging and breakup of droplets are ignored.
(3) Compared with a sneeze, the breath air flow is much weaker and can be ignored.
(4) The thermophysical parameters of droplets are assumed to be constant.
(5) The air is considered to be an ideal gas.
(6) The air flow of the ambient is neglected.

#### 2. Mesh description

**FIG. 3.**
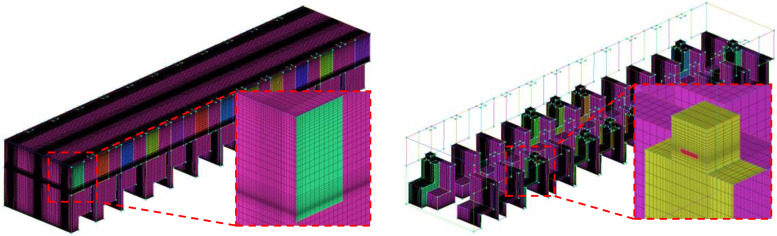
The structured tetrahedral mesh of the computational domain.

In this paper, a structured tetrahedral mesh is constructed in the computational domain. As shown in Fig. 3, the local grids near the wall and the mouth are carefully refined. The grid independent test is conducted in this paper. The velocity magnitude and gauge pressure at the center point of the model (*x *=* *6000 mm, *y *=* *1275 mm, and *z *=* *1000 mm) calculated with four different meshes are compared. According to the results shown in Table II, the structured grid with a mesh number of 2 184 393 is adopted for the simulation.

**TABLE II.** Grid independent analysis with the center point at the coordinate of *x *=* *6000 mm, *y *=* *1275 mm, and *z *=* *1000 mm.

Mesh number	477 557	1 222 080	2 184 393	3 171 447
Velocity (m/s)	0.086 27	0.092 59	0.099 72	0.099 24
Gauge pressure (Pa)	3.7019	3.5832	3.5668	3.5680

#### 3. Numerical solution method and boundary condition

The governing equations are discretized by the finite volume method and solved by the semi-implicit method for pressure linked equation-consistent (SIMPLEC) scheme. The least squares cell-based method is used for gradient discretization, the second-order upwind scheme is used for the momentum equations, and PRESTO! is used for pressure. The total time of the simulation is 100 s. To obtain accurate results for the movement of the droplets, the time step Δ*t* is set to be 0.001 s during sneezing (0–0.12 s) and increased to 0.01 s from 0.12 to 1 s and 0.1 s from 1 to 100 s.

**FIG. 4.**
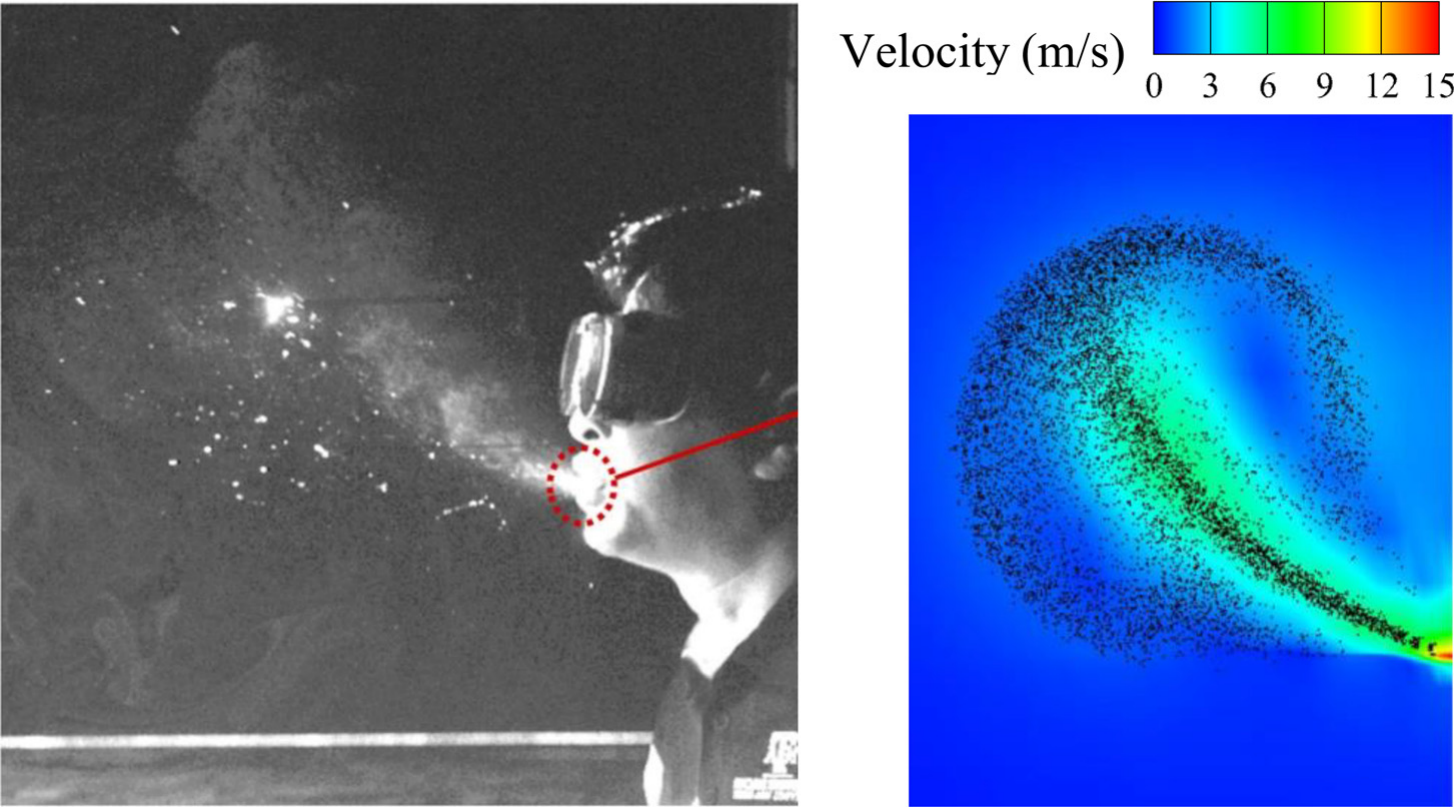
The comparison of droplet distribution between the simulation results and the snap picture in Busco's^13^ work.

Figure 4 compares the droplet distribution of a sneezing between the snap picture in Busco's^13^ work and the simulation results based on our numerical solution method. As seen from the figure, similar droplet distribution can be obtained at the same time of the sneezing (t = 0.19 s in the picture). Therefore, it can be considered that the numerical solution method used in this paper for sneezing simulation is reasonable.

**FIG. 5.**
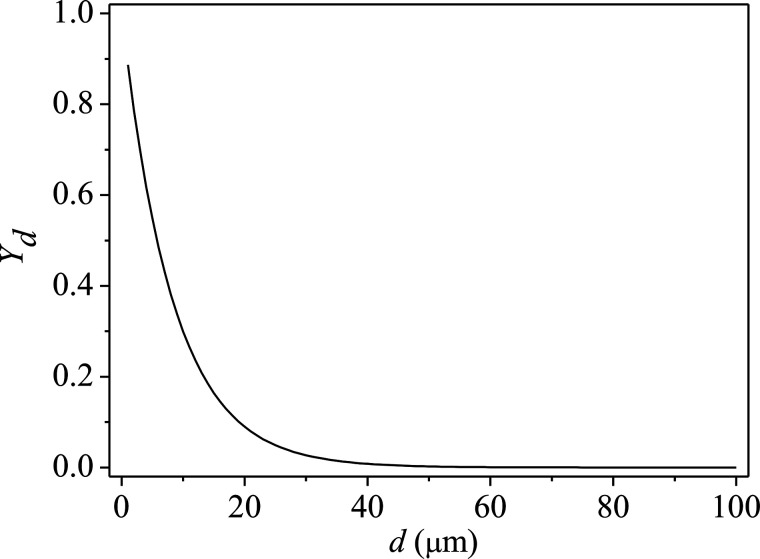
The droplet diameter distribution obeys Rosin–Rammler law.

In this paper, the ambient temperature is set at −4° for the winter influenza season. The temperature of air outside of the air conditioning is 20 °C, and the inlet velocity is 0.78 m/s. The opening window is set as outflow, while the closed windows are set as wall. The surface temperature of a normal person is 36.5 °C, while it is 39 °C for a patient with COVID-19 according to the numerical study from Wu *et al.*^10^ The wall of the bus and the chair are set to be adiabatic. The velocity of air from the mouth during sneezing is set to 50 m/s based on the CFD simulations conducted by Wang *et al.*,^21^ and the temperature is 39 °C, which is the same as the body temperature. Afterward, the mouth is considered to be closed, i.e., the mouth is changed into a wall boundary when sneezing is finished. The droplets coming out of the mouth have a velocity of 50 m/s in 0.12 s, and the diameter distribution of the droplets obeys the Rosin–Rammler distribution law,^22^ which can be expressed as $Y_{d}=e^{-{\left(d/\bar{d}\right)}^{n}}$, where *Y_d_* is the mass fraction of droplets of diameter greater than a droplet diameter, *d* is the droplet diameter, $\bar{d}$ is the mean diameter of droplets, and n is the size distribution parameter. In this paper, the range of droplet diameter is 1–100 *μ*m, and the mean diameter of droplets is 8.3 *μ*m. Therefore, the droplet diameter distribution can be obtained as shown in Fig. 5. The density of droplets is 1100 kg/m^3^, the total number of droplets is 7347, and the total flow rate of droplets is 6.59 × 10^−6 ^kg/s.

## III. RESULTS AND DISCUSSION

To study the effect of the positions of open windows on the spreading of droplets, five cases with different open window configurations are examined. All of the cases simulated in this paper are listed in Table III.

**TABLE III.** The list of the cases conducted in this paper.

	Open window number
Case 1	6a
Case 2	6b
Case 3	5a
Case 4	5b
Case 5	5a, 5b, 6a, 6b, 7a, 7b

### A. Air flow in the bus

**FIG. 6.**
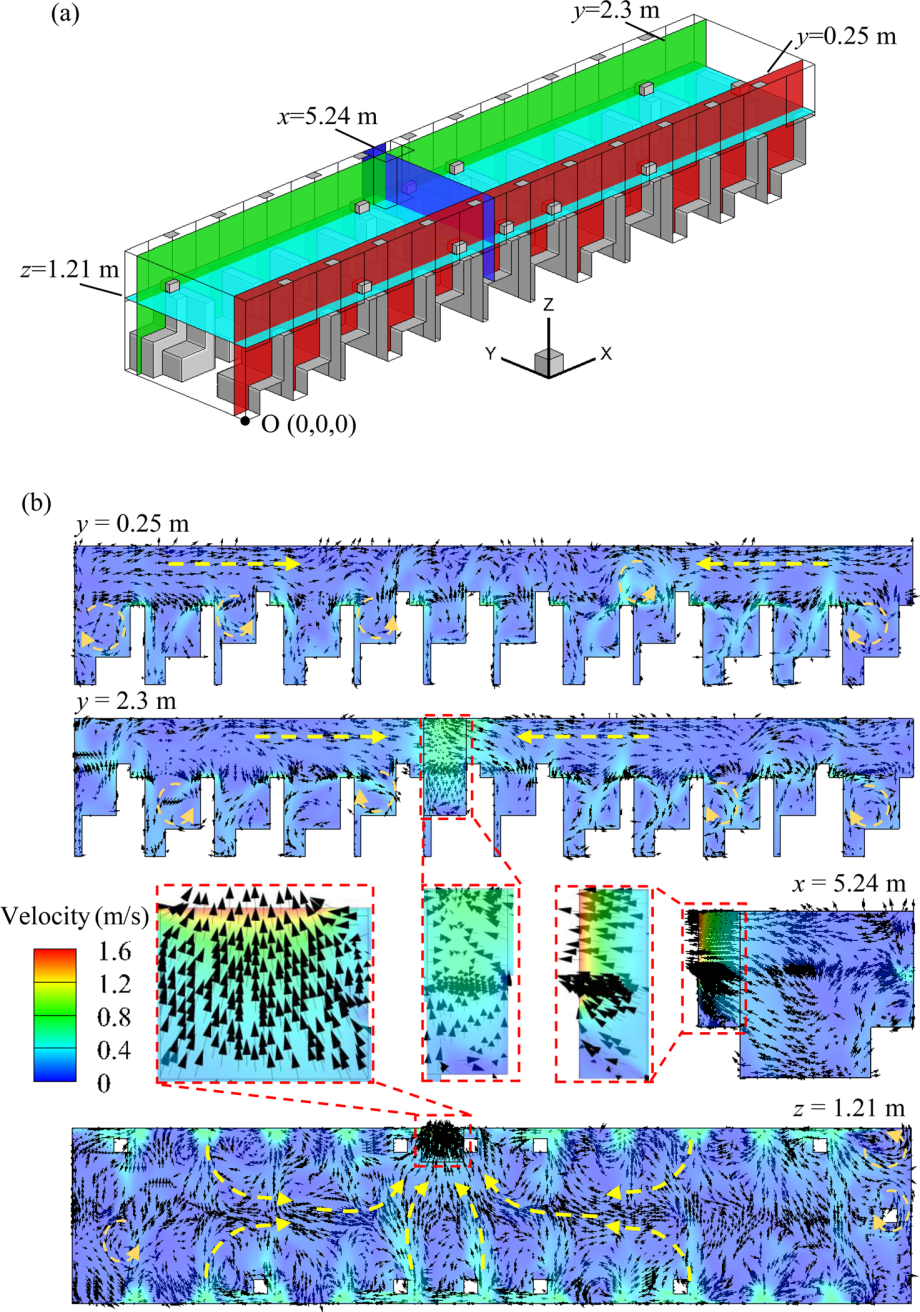

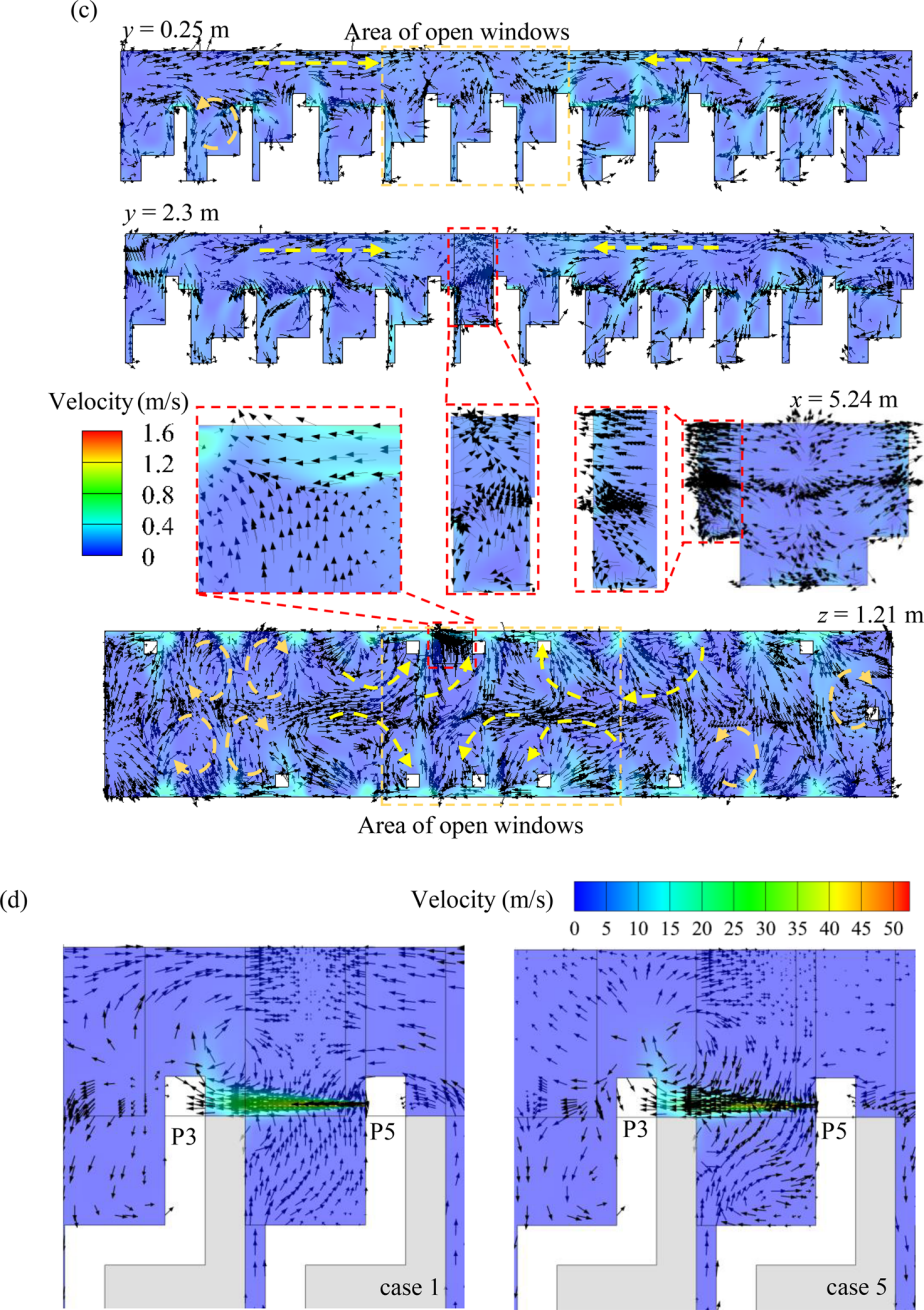
Air flow organization in the bus: (a) position of the sliced planes; (b) flow field of case 1; (c) flow field of case 5; and (d) flow field between P3 and P5 at 0.12 s.

A general perception is that opening a window near an infected person is helpful for reducing the transmission of the virus inside the bus. Therefore, the case in which P5 sits in the sixth row is assumed to be the infected person, and window No. 6a (right next to the infected person) remains open was first simulated. Figure 6 shows the velocity field of several planes sliced [as shown in Fig. 6(a)] from the computational domain. It should be noted that although the passengers are not distributed symmetrically, the flow field of the other three cases (cases 2–4, including the flow direction of the mainstream and independent areas separated by the position of the open window) with one open window is quite similar to that of case 1. Therefore, this type of flow organization is the typical phenomenon for situations with one open window. As shown in Fig. 6(b), the air flow in the bus mainly flows toward the open window. In addition, taking the plane *x *=* *5.24 m as the separating surface, two air mainstreams with opposite flow directions can be identified in the bus. It is not easy for the air from one area to flow into the other area, which infers that there exist two relatively independent spreading regions in the bus. However, several vortices can be observed in the two independent areas [Fig. 6(b)], indicating that the enhanced local spreading of droplets may be caused by the disordered air flow. Figure 6(c) shows the air flow field in case 5. As shown in the figure, two independent areas with a smaller range and more vortices can be identified, in which the air mainly flows toward the zone of open windows. In addition, different from case 1, once the air flows into the area with open windows, the flow becomes multidirectional as six windows are open. In other words, the air flow is more turbulent when opening multiple windows. This flow organization may promote virus spreading in this area, which will be further discussed in Sec. III B. As shown in Fig. 6(d), the airflow from sneezing has already reached the person in the front row in 0.12 s due to the high initial velocity. As a result, the person in the front row has been basically shrouded in the aerosol which contains a considerable number of particles, leading to a high risk of virus infection. Therefore, avoiding sitting in the row in front of the infected person is strongly recommended. In addition, this serious situation indicates the importance of wearing facemask, which is not only preventing from inhalation of aerosol with virus but also stopping the spread of virus owing to the sneezing.

### B. Droplets spreading with air flow

**FIG. 7.**
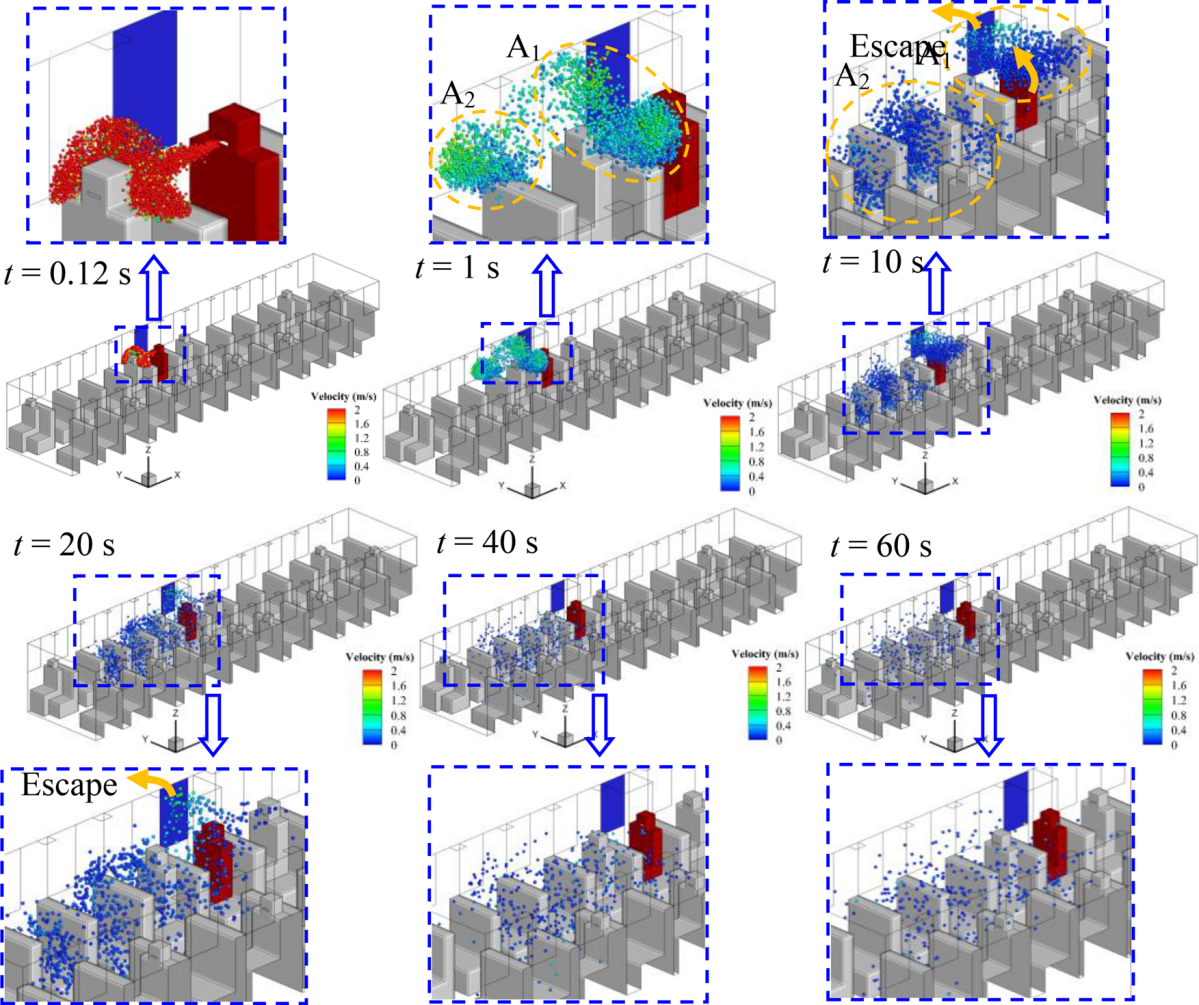
Spreading process of droplets in case 1.

**FIG. 8.**
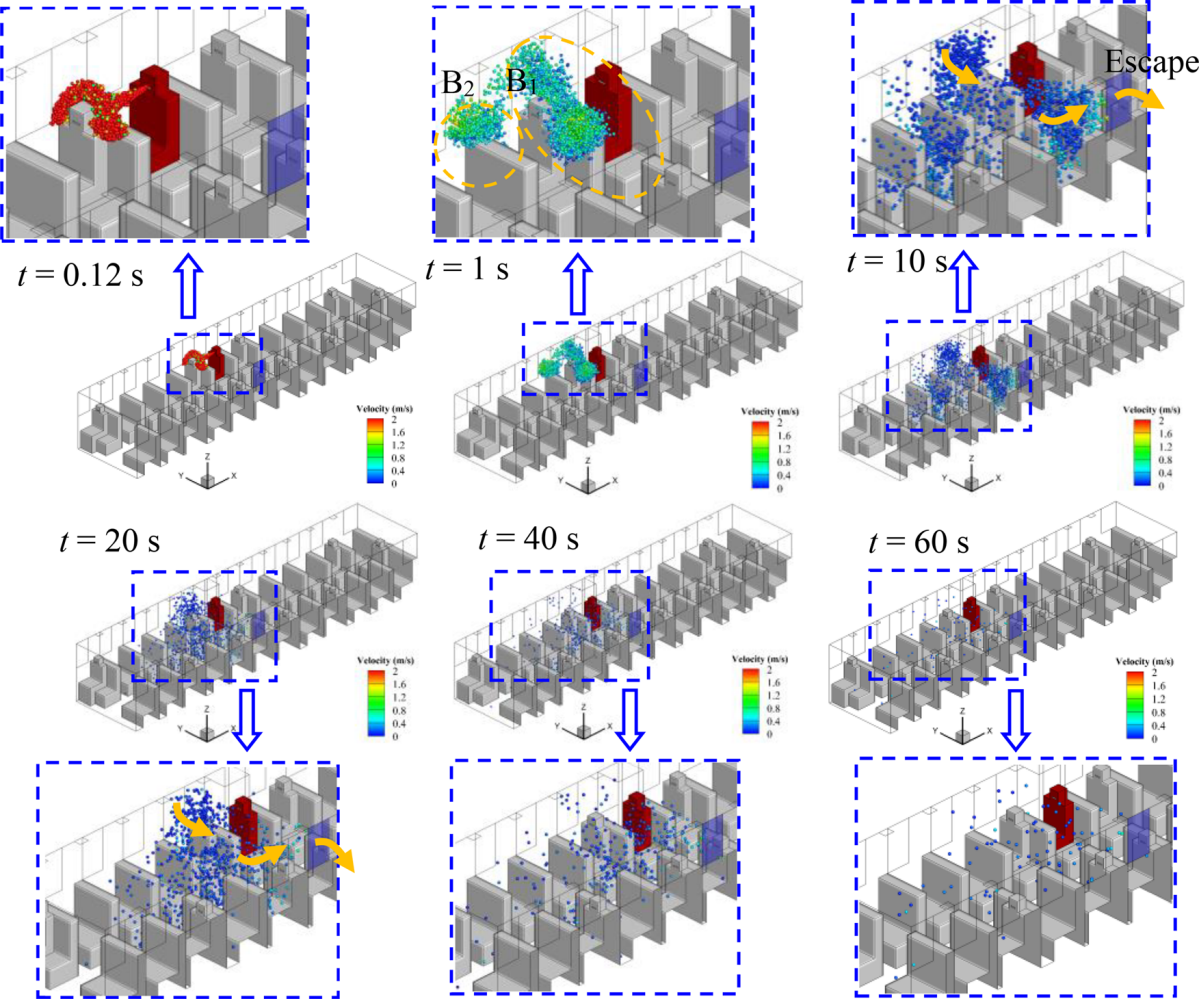
Spreading process of droplets in case 2.

**FIG. 9.**
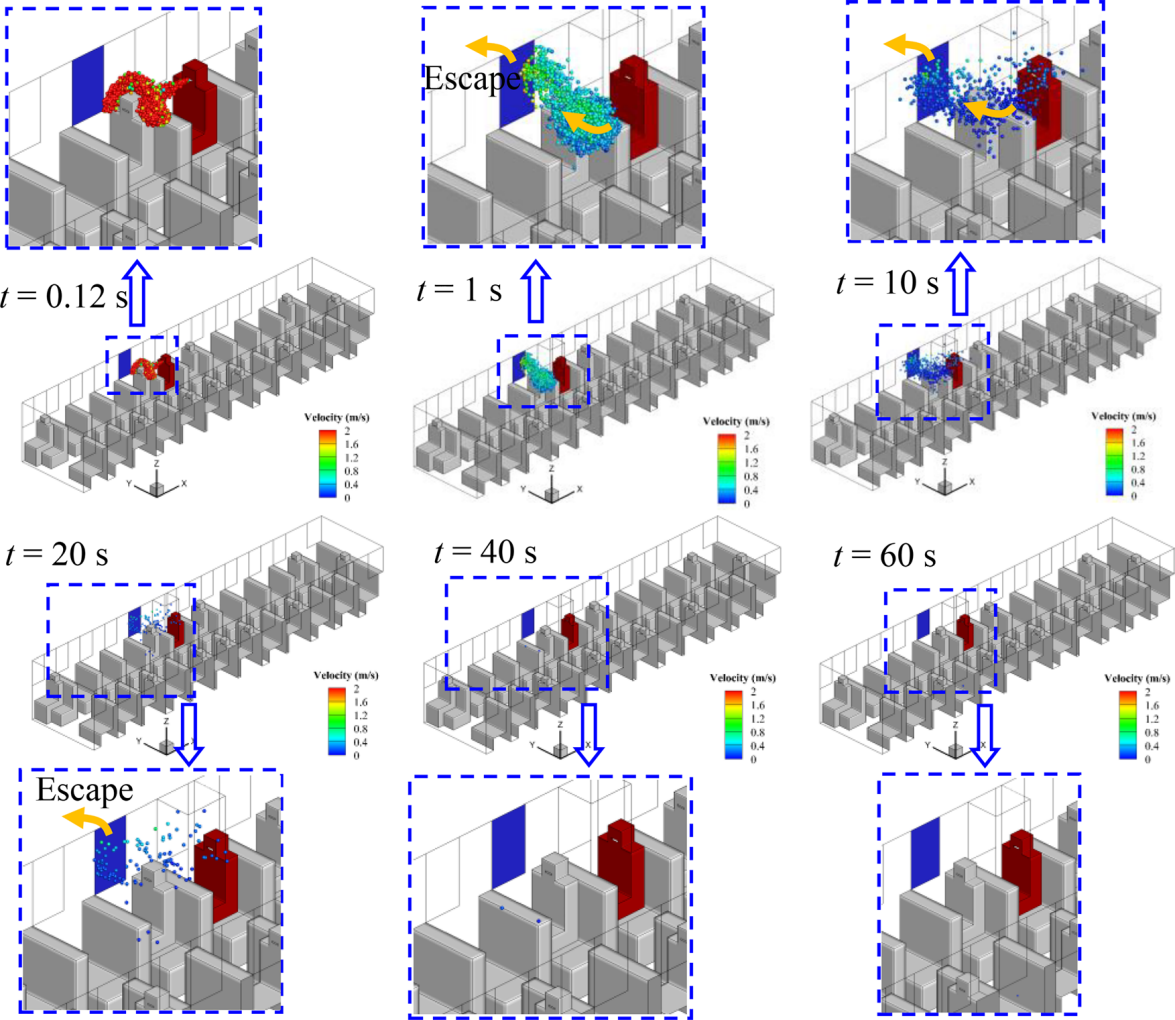
Spreading process of droplets in case 3.

**FIG. 10.**
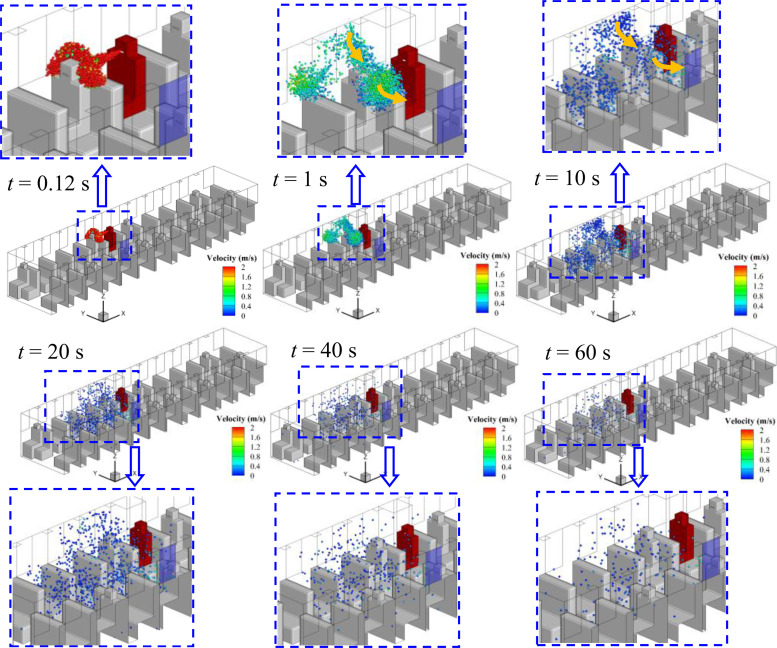
Spreading process of droplets in case 4.

**FIG. 11.**
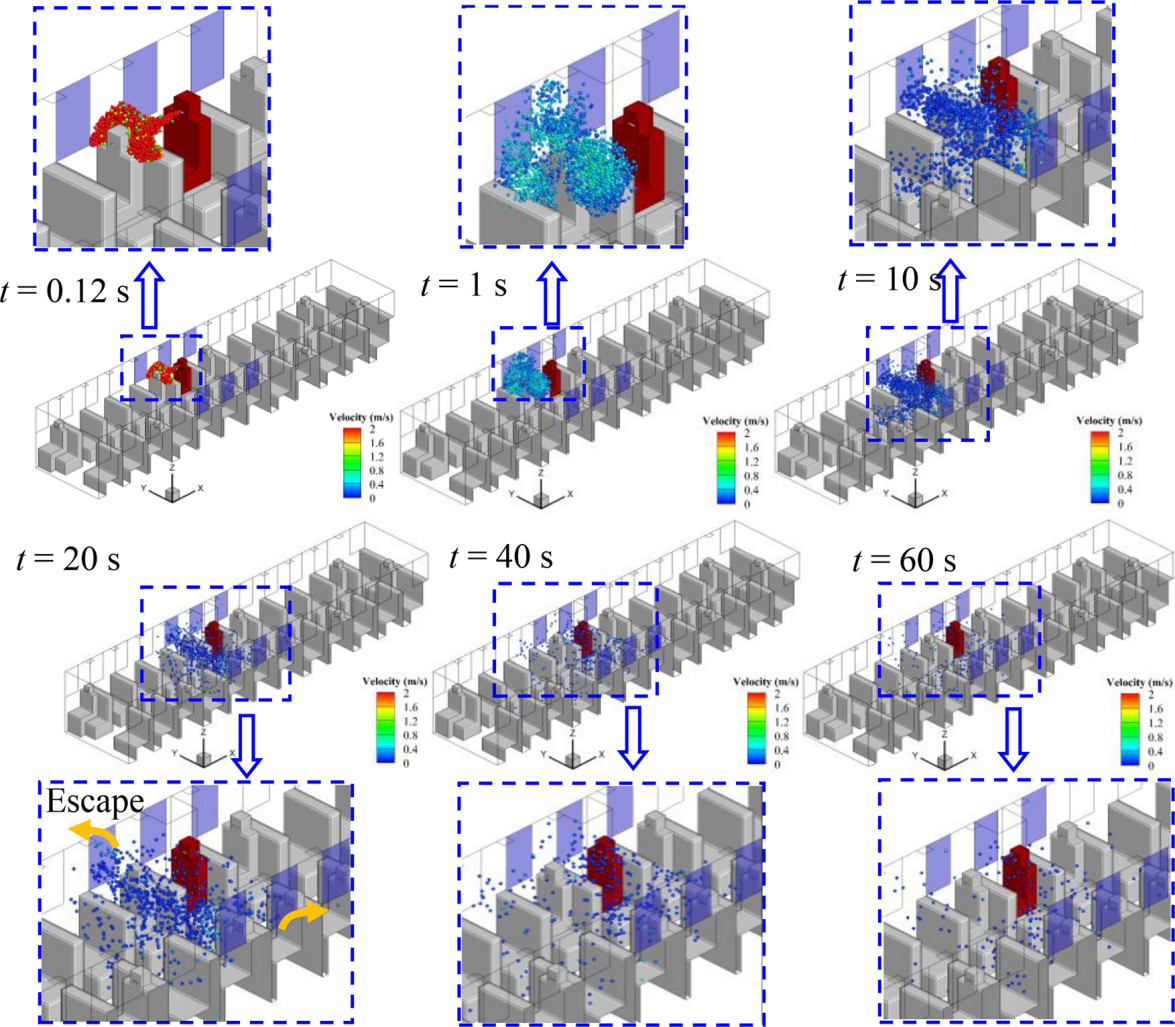
Spreading process of droplets in case 5.

Considering the relative position of the infected person and the window, the spreading processes of droplets in cases 1–5 are quite different, although the flow field is quite similar. The evolution of droplet distributions with time (*t *=* *0.12, 1, 10, 20, and 40 s) for different cases is compared in Figs. 7–11. The open window is colored blue, and the infected person is colored red. As seen in the figures, the droplets have already reached the passenger in front of the infected person at 0.12 s and shrouded this passenger for a long time in all cases. Therefore, it is strongly recommended that passengers sit dispersedly as much as possible while taking public transportation.

#### 1. Case 1—Window No. 6a

The No. 6a window is right beside the infected person. As shown in Fig. 7, two agglomerations of droplets marked A_1_ and A_2_ can be seen in Fig. 7(b). The droplets in agglomeration A_1_ move back to the infected person along with the air mainstream, and the spreading range is limited to the infected person and escapes from the open window in 40 s. Therefore, the movements of droplets in agglomeration A_1_ show a limited effect on the spread of the virus. However, the other portion of droplets in agglomeration A_2_ spread quickly in the front part of the bus after it moves to the second row in front of the infected person in 1 s. As shown in Fig. 7(c), the droplets of A_2_ sufficiently filled the space in front of the infected person 20 s after sneezing. In addition, little reduction of the droplets can be observed even if the spreading process has continued for 60 s. As a result, a high risk of being infected with COVID-19 by aerosol transmission in the front part of the bus could be caused by taking the strategy of opening window No. 6a, which is beyond our general expectation.

#### 2. Case 2—Window No. 6b

As shown in Fig. 8, window No. 6b is on the other side of the aisle. When the infected person sneezes, two agglomerations of droplets (B_1_ and B_2_) can also be seen after 1 s. The droplets of the B_1_ agglomerate, which is the main part of droplets from sneezing, move across the aisle to the other side of the bus due to the air flow toward the opening window, and then escape from window No. 6b. As shown in Fig. 8(c), passengers P3 and P4 may have a higher chance of being infected than other people and should be treated as people who are in close contact with the infected person because they are on the flow path of air and exposed to droplets of B_1_ directly for a long time. The B_2_ agglomeration moves to the front part of the bus and then disperses. However, the dispersion range is slightly smaller than case 1 as affected by the reverse airflow. In addition, the decrease in droplets in the air over time is more obvious when compared with case 1. Therefore, this strategy shows better performance in weakening the spread of the virus in the bus.

#### 3. Case 3—Window No. 5a

Figure 9 shows the droplet spreading process when window No. 5a opens, and an encouraging result can be observed in this case. As shown in the figure, the droplets escape from the bus through window No. 5a with the air flow soon after the droplets reach passenger P3. As a result, the range of droplet spreading is quite limited, which is beneficial for minimizing the aerosol transmission of COVID-19. In addition, Fig. 9(e) shows that very few droplets remain in the bus 40 s after sneezing. Therefore, except for passenger P3, the risk of exposure to COVID-19 by aerosol transmission will be greatly reduced for other people in the bus.

#### 4. Case 4—Window No. 5b

Different from the encouraging situation in case 3, when the opening window has changed to window No. 5b, the spreading of droplets is alarming. As shown in Fig. 10, the droplets have to move across the aisle before escaping from window No. 5b. During this movement, the droplets disperse to the carriage under the action of the vortex and fill the front half of the bus in 20 s. Furthermore, similar to cases 1 and 2, there remain a considerable number of droplets in the air after 60 s. Therefore, keeping window No. 5b open is adverse for the suppression of droplet spreading, and the air will be polluted in a large range by virus-containing droplets.

#### 5. Case 5—Multiple windows

Keeping multiple windows open usually results in good air circulation but will cause obvious air turbulence (as shown in Fig. 6), which may lead to the rapid spread of droplets. With this concern, the case of opening multiple windows (Nos. 5a, 5b, 6a, 6b, 7a, and 7b) is simulated in this paper. It can be observed in Fig. 11 that most of the droplets are restricted in the area of opened windows and escape from window Nos. 5a and 5b. In addition, as seen in Fig. 11(c), the droplets spread to the other side of the bus in 10 s, indicating that the spreading is accelerated due to the turbulent flow of air. Furthermore, the droplets remaining in the bus after 60 s are approximately the same as in cases 2 and 4, which indicates an unsatisfactory performance of minimizing the spreading of droplets.

It can be seen from Figs. 7–11 that once the droplets go into the two independent areas [shown in Fig. 6(b)] in the bus, the droplets will float in the air for a long time and eventually diffuse into the entire carriage under the promotion of vortex. As a result, the cross infection of virus is almost inevitable in the bus, every person in the carriage is in danger of being infected, especially for the person in the front part of the bus.

### C. Statistics for droplet spreading and deposition

**FIG. 12.**
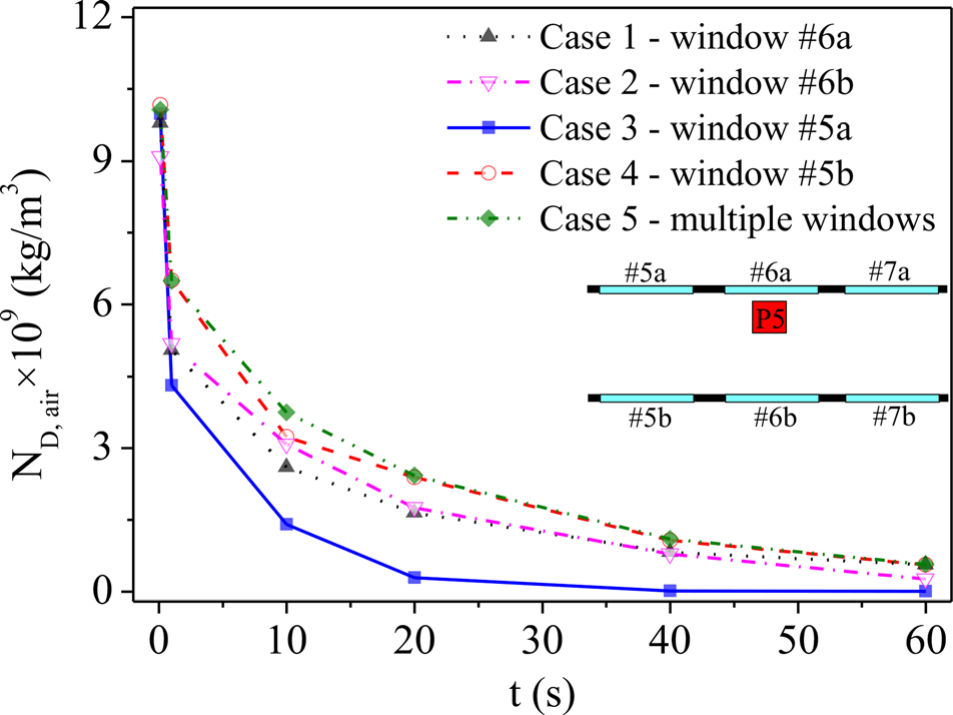
The variation in droplet concentration in air as a function of time.

Figure 12 shows the concentration of the droplets in the space of the bus (N_D,air_) at different times for each case. As seen in the figure, N_D,air_ of case 3 shows the fastest decreasing speed and the largest reduction degree, which becomes almost zero after 40 s. Case 5 shows the least satisfying performance for minimizing the spreading of droplets in the bus because it has the lowest decreasing trend and the highest droplet concentration after 60 s. A similar situation can also be concluded for case 4 with the same variation in droplet concentration as a function of time in this case, which is almost the same as that in case 5. A higher decreasing rate of N_D,air_ can be observed in case 1 when compared with case 2, as many droplets escaped from the opening window in the early stage of spreading in case 1 (agglomeration A_1_ shown in Fig. 6). However, the N_D,air_ of case 1 is higher than that of case 2 after 30 s, as the droplets in another agglomeration (A_2_ shown in Fig. 6) eventually spread into the air and remain in the bus for a long time. In conclusion, from the view of aerosol transmission, the strategy of opening window No. 5a is most beneficial in limiting the spreading range of droplets and reducing the risk of virus transmission to the other passengers in the same bus.

**FIG. 13.**
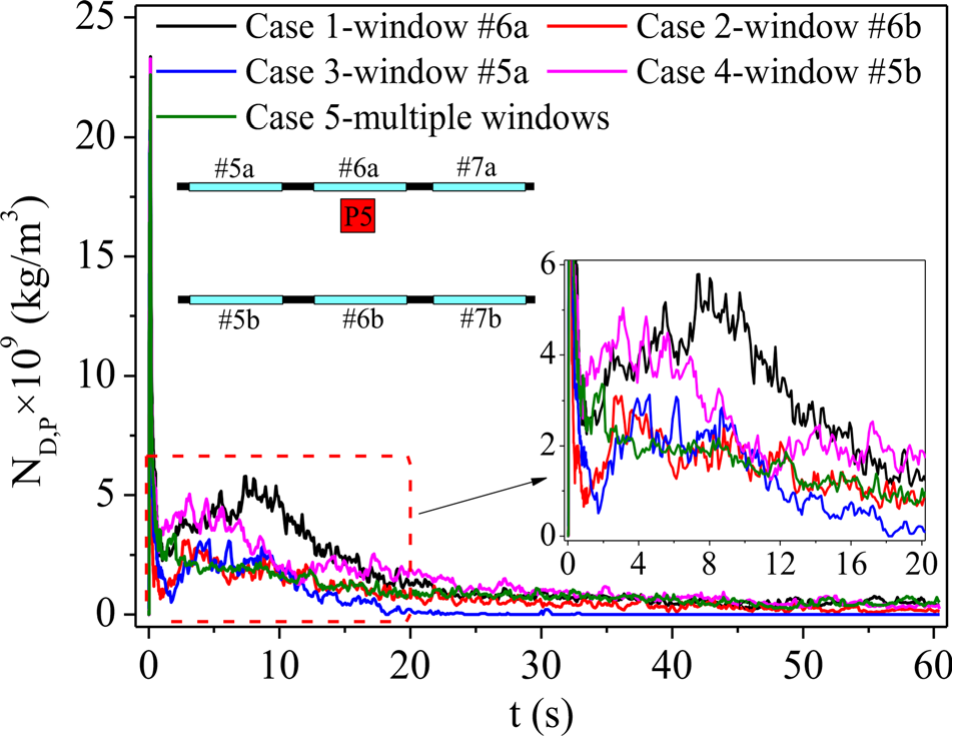
Transient droplet concentration on the surfaces of passengers.

For the droplets ejected by sneezing, except for the portion that escapes through the window with air flow shown in Figs. 7–11, some of them will adhere to the surfaces of passengers. Potential contact transmission will then be caused by the viruses that remain on those surfaces with the deposition of droplets. Therefore, the transient droplet concentration of the passenger surfaces (N_D,P_) for each case is counted, as shown in Fig. 13. It can be seen in the figure that there is a drastic increase in N_D,P_ at the beginning for all cases due to the very dense droplets near the passenger in front of the infected person. Then, N_D,P_ falls sharply after 1 s as the droplets start to diffuse in the air. It can be seen in the figure that the N_D,P_ of case 1 possesses the highest value due to the most diffused droplets with quite high droplet concentrations in the air. As shown, a peak value of N_D,P_ in approximately 10 s can be seen for case 1, indicating that passengers P3 and P5 have been surrounded by droplets (as shown in Fig. 9). For case 4, the peak value appears after approximately 4 s. Combined with Fig. 10, this is just the moment that the agglomerations of droplets move to the place where passenger P6 sits before totally spreading. It should be noted that a higher droplet concentration means a higher risk of COVID-19 infection. In contrast, no obvious peak value can be seen for cases 2, 3, and 5, indicating a lower risk of infection compared with cases 1 and 4. However, as shown in Fig. 12, the droplet concentration in the air for case 5 (multiple windows) always is the highest among all the cases, which means that the droplets cannot be taken out from the bus effectively by the air flow. In addition, it can be seen from Fig. 13 that the N_D,P_ remains steady for a long time in case 5, indicating continuous attachment of droplets on the passengers. Therefore, by combining Figs. 12 and 13, it can be seen that the droplets is promoted to spread around due to the chaos flow caused by opening multiple windows, and then the droplets remaining in the bus will gradually fall on the passengers. As a result, a number of droplets deposited on the surface gradually accumulate, and the risk of contact transmission cannot be neglected under case 5. In contrast, the N_D,P_ of case 3 decreases to almost zero soon after 20 s, which means that few droplets remain in the air. The aerosol transmission, as well as the contact transmission, can be ignored under this situation except for P3.

## IV. CONCLUSIONS

Understanding that the spreading process of droplets is necessary for minimizing aerosol transmission in an enclosed bus during the waiting period is important. In this paper, the spreading process of droplets with indoor air flows under various cases with different positions of the opening window is analyzed. The changes in droplet concentration in air with time under different cases are compared and discussed. In addition, the transient droplet concentrations deposited on the passengers under different cases are also compared to study the potential possibility of contact transmission in the bus. The conclusions are as follows:

(1) Keeping the window open next to the infected person (window No. 6a in this paper) unexpectedly shows an unsatisfactory performance of limiting the droplet spreading range and reducing the droplet concentration. A high risk of being infected with COVID-19 by aerosol transmission in the front part of the bus can be caused by opening window No. 6a. In contrast, when the window in front of the infected person (window No. 5a in this paper) opens, most of the droplets have escaped through the opened window with the air flow in a short time, which is beneficial for minimizing the aerosol transmission in the bus.
(2) The spreading of droplets is accelerated due to turbulence when opening multiple windows, the spreading range is expanded, and the residence time of droplets in the bus is extended. Therefore, the effect of opening multiple windows on the removal of droplets in a bus is not obvious.
(3) Avoiding sitting in the row in front of the infected person is strongly recommended, as there will be a high concentration of droplets when the infected person sneezes in less than 1 s, which causes a high risk of infection by aerosol transmission and subsequent contact transmission due to the deposition of droplets.
(4) The risk of getting the virus from potential contact transmission should not be ignored for all cases except for case 3 since considerable droplets diffuse into the air. For case 3, few droplets remain in the air soon after sneezing, so aerosol transmission and contact transmission can be ignored.

## Data Availability

The data that support the findings of this study are available from the corresponding author upon reasonable request.

## NOMENCLATURE

*C_μ_*: Turbulence fields
*C*_1_: Constant, max [0.43, *η*/(*η* + 5)]
*C*_1__*ε*_: Constant, 1.44
*C*_2_: Constant, 1.9
*D*: Distance between rows (m)
*d*_p_: Thickness of passenger (m)
*d*_seat_: Total length of seat (m)
$\vec{F}$: Acceleration caused by additional forces (m^2^/s)
*F*_D_: Reciprocal of the droplet or particle relaxation time (1/s)
*G_b_*: Turbulence kinetic energy due to the buoyancy force
*G_k_*: Turbulence kinetic energy due to the mean velocity gradients
*H*: Height of vehicle (m)
*h*_p_: Height of passenger (m)
*h*_seat_: Height of seat (m)
*h*_win_: Height of window (m)
*k*: Turbulent kinetic energy
*L*: Length of vehicle (m)
*l*_seat_: Length of seat surface (m)
*M*_t_: Turbulent Mach number
$\vec{u}$: Air velocity (m^2^/s)
${\vec{u}}_{p}$: Single droplet velocity (m^2^/s)
*W*: Width of vehicle (m)
*w*: Width of aisle (m)
*w*_p_: Width of passenger (m)
*w*_seat_: Width of seat (m)
*w*_win_: Width of window (m)

**Greek symbols**
ε: Dissipation rate
*μ*_t_: Turbulent viscosity (m^2^/s)
ρ: Air density (kg/m^3^)
ρ_p_: Droplet density (kg/m^3^)
σ_k_: Turbulent Prandtl numbers

**Subscripts**
b: Buoyancy force
p: Passengers in the vehicle
seat: Seats in the vehicle
win: Windows in the vehicle
